# Fetal ECG-based analysis reveals the impact of fetal movements and maternal respiration on maternal-fetal heart rate synchronization

**DOI:** 10.1371/journal.pone.0312310

**Published:** 2024-12-02

**Authors:** Amna Samjeed, Maisam Wahbah, Leontios Hadjileontiadis, Ahsan H. Khandoker

**Affiliations:** 1 Department of Biomedical Engineering and Biotechnology, Khalifa University, Abu Dhabi, United Arab Emirates; 2 College of Engineering and Information Technology, University of Dubai, Dubai, United Arab Emirates; 3 Healthcare Engineering Innovation Group (HEIG), Department of Biomedical Engineering and Biotechnology, Khalifa University, Abu Dhabi, United Arab Emirates; 4 Department of Electrical and Computer Engineering, Aristotle University of Thessaloniki, Thessaloniki, Greece; Polytechnic University of Marche: Universita Politecnica delle Marche, ITALY

## Abstract

Identifying and understanding prenatal developmental disorders at an early stage are crucial as fetal brain development has long-term effects on an individual’s life. The maturation of the fetal autonomic nervous system (ANS) is believed to influence the coordination and direction of maternal-fetal heartbeat synchronization. Fetal behavioral states (FBSes) include quiet sleep (1F), active sleep (2F), quiet awake (3F), and active awake (4F). In this study, the focus is on fetal movements, leading to the grouping of 1F and 3F into a quiet state, while 2F and 4F are combined to form an active state. Thus, the FBSes discussed in this article consist of fetal quiet and active states. Here, we explore the relationship between FBSes and the coupling of maternal and fetal heartbeats. We also seek to understand how maternal breathing patterns influence this coupling while considering FBSes. The study involved 105 healthy fetuses with gestational ages (GA) from 20 to 40 weeks. Non-invasive electrocardiogram (ECG) signals were recorded for 3 to 10 minutes. The ECG samples were separated into three gestational groups (Early: 16 ≤ GA < 25, Mid: 25 ≤ GA < 32, and Late: 32 ≤ GA < 40 weeks). Maternal respiration rate and coupling strength parameters were calculated for various maternal-fetal heartbeat coupling ratios. The findings of the study indicated that FBSes influenced maternal-fetal HR coupling strength during late gestation but not during early and mid-gestation. The changes in maternal-fetal HR synchronization or communication as gestation progresses occur in both FBSes. Furthermore, we noticed a significantly higher level of maternal-fetal heartbeat synchronization during periods of higher respiratory rates when the fetus was in a quiet state. These results emphasize how FBSes impact the synchronization of maternal-fetal HR and contribute to the understanding of fetal growth and health.

## Introduction

The fetal neurological system undergoes gradual development over the first, second, and third trimesters of pregnancy [1, 2]. The growth of spinal cord synapses commences during the seventh week [3], coinciding with the onset of electrical activity in the brain, facilitating spontaneous movements. The fetus exhibits more noticeable involuntary movements at the onset of the second term of pregnancy, as identified through ultrasound imaging [4]. These activities encompass yawning, sucking, and other coordinated movements as the brain undergoes continuous development each week. However, it is important to note that the initial signs of vital function control, such as regulating heart rate (HR) and breathing, do not become apparent until the latter part of the second trimester, till the brainstem fully matures [5, 6]. Furthermore, the cerebral cortex takes shape during the third trimester, enabling the emergence of voluntary actions like thinking and experiencing emotions while advancing the development of motor skills involving various body parts and senses as pregnancy progresses towards its end [6, 7].

Fetuses undergo continuous developmental changes during pregnancy, such as circadian rhythm establishment and autonomic nervous system (ANS) maturation. These changes manifest in different fetal behavioral states (FBSes), such as quiet sleep (1F), active sleep (2F), quiet awake (3F), and active awake (4F) [8]. Infrequent gross body movements characterize states 1F and 3F, and they are together called a quiet state. On the other hand, states 2F and 4F involve regular gross body movements; they are together called active states [9]. FBSes can only be classified into 1F, 2F, 3F, and 4F after 32 weeks of gestation [10]. Prior to this period, FBSes can only be categorized into fetal quiet state and fetal active state. Since our study utilizes data from 20 to 40 weeks of gestation, we have chosen to classify FBSes into fetal quiet state and active state instead of 1F, 2F, 3F, and 4F. Heart rate variability (HRV) and ANS activity vary according to the FBSes, which may affect the coordination and synchronization of heart rate patterns (HRP) between the mother and child [11]. Non-invasive electrocardiogram (ECG) is one of the methods to obtain the HRV and HRP accurately from the early fetal gestation itself.

A measure of maternal-fetal HR coupling (MFHRC) refers to the degree to which maternal and fetal HR are coordinated and synchronized [12]. It indicates the functional interaction between the maternal and fetal cardiovascular systems and can be influenced by several factors, which may include the FBSes [13]. The relationship between MFHRC and these states can provide valuable information regarding fetal well-being, the development of the ANS, and the integrity of the cardiovascular system during pregnancy [14].

The mother’s physical condition plays a major role during pregnancy in determining the fetal environment and development. Despite this, little information is available regarding maternal perceptions of the condition of the fetus. The literature suggests a physiological interaction between the mother and the fetus, with several studies suggesting a correlation between maternal and fetal conditions based on fetal HR. In particular, there is evidence that changes in fetal HR and HRV are associated with variations in maternal arterial oxygen content [15], maternal hypothermia [16], and maternal exercise [17]. A strong correlation exists between the mother’s physical and psychological well-being and the fetal HR throughout the pregnancy [18]. In some studies, maternal stress has been shown to increase fetal HR [19], whereas nocturnal activities are known to decrease maternal and fetal HR [20]. Furthermore, maternal exercise that increases HR can result in hypoxia in the fetus [21]. It was found that changes in maternal respiration affect synchronization [22] since rapid breathing causes MFHRC. In contrast, slower breathing produces greater vagal tone and beat-to-beat differences, reducing the coupling effects.

There is still a need to explore the underlying mechanisms through which MFHRC is affected by FBSes. It is hypothesized that variations in the activity of the fetal ANS, including sympathetic modulation and parasympathetic modulation, are crucial to this process [23]. Specific ANS activation patterns are associated with FBSes, which affect HRV and coordination. Other factors, such as fetal movements and changes in blood flow distribution during various FBSes, may also influence the observed changes in MFHRC.

A thorough understanding of how MFHRC is influenced by FBSes has significant implications for clinical practice. An abnormal HR phase coupling during specific FBSes may indicate compromised fetal health, cardiac function, or ANS development. These changes should be monitored and analyzed to help detect fetal distress early, guide interventions and improve perinatal outcomes.

Various methodological approaches have been proposed to identify maternal-fetal heart rate coupling (MFHRC). These methods include synchrograms and phase coherence indices [22, 24–26], deep coherence [27], joint symbolic dynamics [6, 7], cross-correlation [28, 29], bivariate phase rectified signal averaging [30], Granger causality and partial directed coherence [31], and transfer entropy [13, 18]. Based on the review [14] done recently, most of the studies used synchrograms and phase coherence indices under various conditions like maternal exercise, GA progress, and maternal respiration rate. However, none of the studies considered FBSes. Factors like fetal movements and alterations in blood flow distribution during various FBSes could contribute to the variations in MFHRC.

In this study, we explore the impact of FBSes on MFHRC using synchrograms and phase coherence indices. We also analyzed the relationship between maternal respiration and maternal-fetal HR synchronization, taking FBSes into account. The study involved 105 healthy fetuses with gestational ages (GA) from 20 to 40 weeks. ECG signals were recorded for 3 to 10 minutes. Maternal respiration rate and coupling strength parameters were computed for various maternal-fetal heartbeat coupling ratios.

The rest of the paper is organized as follows: First, the ‘methodology’ section discusses the data collection and analysis techniques used. Then, the ‘result’ section analyzes the data and finds any significant patterns or relationships. Following the ‘results’ section, the ‘discussion’ section interprets the findings from the results and clinical applications for the same. Finally, the ‘conclusion’ section summarizes the major findings of the study.

## Materials and methods

### Subjects

A total of 105 fetuses with GA spanning from 20 to 40 weeks were included in the article. Non-invasive ECG signals were recorded for 3 to 10 minutes while pregnant mothers were lying on their backs. The recorded fetal ECG signals were then categorized into three groups based on GA: Early: 16 ≤ GA < 25 weeks, Mid: 25 ≤ GA < 32 weeks, and Late: 32 ≤ GA < 40 weeks. The data for this study were obtained from three institutions, i.e. Kanagawa Children’s Medical Center (16 subjects, 15%), Tohoku University Hospital (75 subjects, 71%), and Children’s National Hospital in the United States (14 subjects, 14%). The research protocols received approval from the Tohoku University Institutional Review Board (IRB: 2015-2-80-1) and the Children’s National Hospital IRB committee, along with the necessary institutional agreements. Written informed consent was obtained from all participants.

### ECG signal processing

Twelve electrodes were strategically placed on the maternal abdomen to capture the signals. Specifically, one of the electrodes was positioned at the right thoracic region and another on the back, and the remaining ten electrodes were placed on the mother’s abdomen. The signals were sampled every 1 millisecond (1 kHz sampling rate) with a 16 bit resolution. The recordings were obtained while the participant was in a supine position. To extract the fetal ECG from the composite abdominal signal, a combination of techniques called maternal ECG cancellation and blind source separation with a reference signal, as mentioned in the previous study [32]. A more detailed description of the experimental setup can be found in a previous study [33]. In summary, the subtraction of the maternal ECG component involved a linear combination of mutually orthogonal projections of the heart vector. Blind source separation was achieved through a neural network method, iteratively calculated from reference signals resembling the target signal.

**Fig 1:**
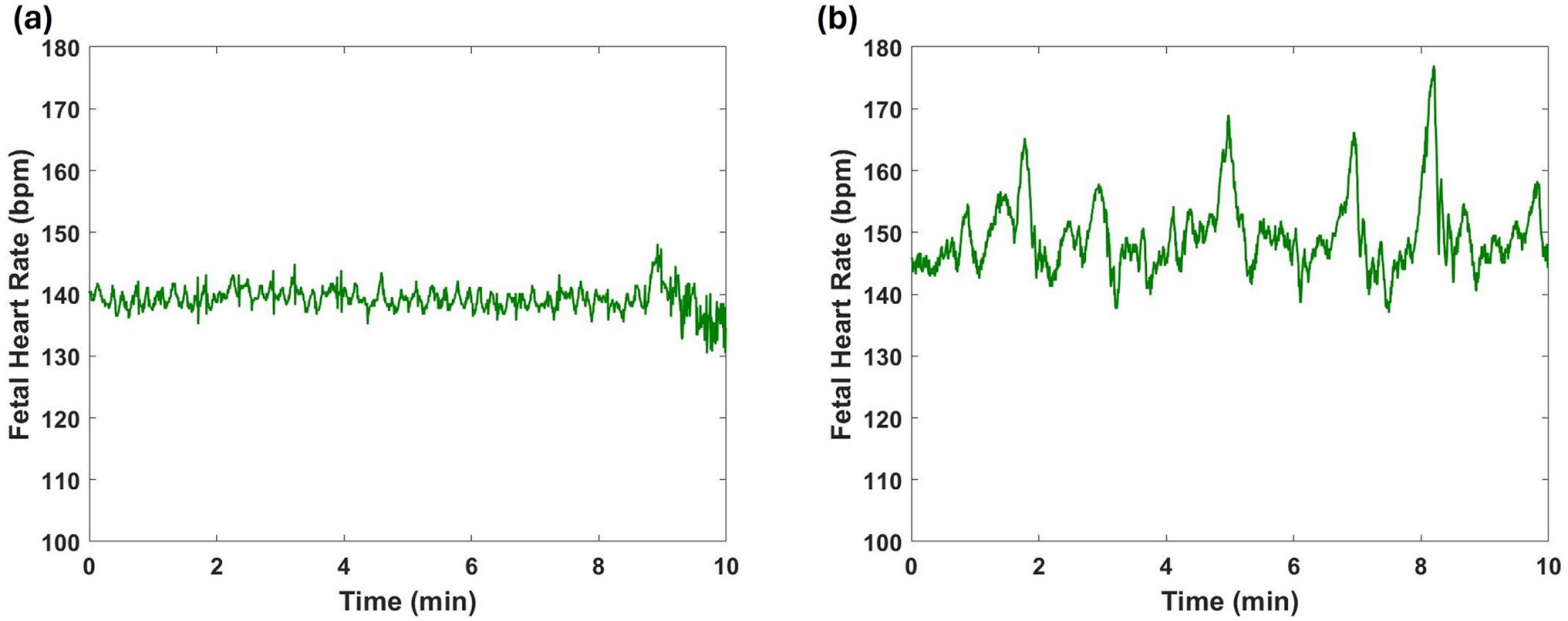
Examples of fetal heart rate (HR) time series signals: (a) Quiet state, and (b) Active state.

After separating fetal and maternal ECG, baseline noise is removed using a high pass filter with a cutoff frequency of 5 Hz. Subsequently, the ECG signals were normalized by dividing each signal by its maximum amplitude value, which corresponded to the highest R peak amplitude value. Then, a custom MATLAB routine program was used utilizing a function called findpeaks for identifying the locations of fetal and maternal R peaks. The findpeaks function identifies peaks using a threshold value, which in this case was adjusted according to the varying amplitudes of the R peaks among different subjects [34, 35]. This function yields the positions of the detected peaks along with their corresponding amplitudes. The identified R peaks were visually confirmed to ensure that the function accurately detected only the R peaks. Finally, the differences between R wave locations were calculated to determine the RR interval. The least window size for separating fetal quiet and active states was 3 minutes [36]. So, in this work, ECGs with a duration of 3 min were segmented from the original recordings. All considered samples in the performed analysis had a unified recording length of 3 min. Each of these 3 minute segments serves as an individual sample dataset, and all the feature extraction procedures described in the subsequent subsections are applied to these segments. Fig 1a shows an example of a fetal HR time series signal during a quiet state, while Fig 1b illustrates an example of a fetal HR time series signal during an active state.

### Phase coupling

The phase coherence method assessed the synchronization or coupling of fetal and maternal ECGs [37]. The instantaneous phase time series (*φ*) was obtained as follows:

$$\begin{array}{c} \varphi \left(t_{k}\right)=\frac{2\pi (t-t_{k})}{t_{k+m}-t_{k}}+2\pi k, \end{array}$$ (1)

where *t* represents the time instances corresponding to R-peaks in the fetal ECG signals, *t*_*k*_ represents the time instances corresponding to R-peaks in the maternal ECG signals, range of *k* is from 1:(Total number of maternal heartbeats-1), and *m* represents the count of maternal heartbeats.

The relative phase Ψ(*t*_*k*_) for the maternal ECG signal within the time window of *t*_*w*_ is

$$\begin{array}{c} \Psi \left(t_{k}\right)=\frac{\varphi (t_{k})\;mod\;2\pi }{2\pi }. \end{array}$$ (2)

where *t*_*w*_ is the time of 70 maternal heartbeats, and the range of *k* is from 1:69 [26].

After calculating the relative phase Ψ(*t*_*k*_), the phase coupling index (λ(*t*_*k*_)) [38] was computed as follows:

$$\begin{array}{c} \lambda \left(t_{k}\right)\;=\;\|\frac{1}{N}\;\sum_{j=k-N/2}^{k+N/2}e^{i\;\Psi (t_{j})}{\|}^{2}, \end{array}$$ (3)

where *N* represents the total number of maternal heartbeats that occur within the time window and was set to 70, while λ(*t*_*k*_) ranges from 0 to 1, with 1 indicating the highest synchronization. In this study, we computed the values of Ψ(*t*_*k*_) and λ(*t*_*k*_) for various maternal-to-fetal heartbeat ratios denoted as [*m:n*], where *n* signifies the count of fetal heartbeats. The selected [*m:n*] ratios included [1:2], [1:3], [2:3], [2:4], [3:4], and [3:5]. The coupling ratios of [1:2], [2:3], and [3:5] in each maternal-fetal segment were reported to be major in the previous article [27]. In addition, ratios of [1:3], [2:4], and [3:4] were reported to occur less. However, they played a role in fetal age estimation. Moreover, the previous study [26] showed that the maternal-fetal heart rate coupling ratios ([1:2], [1:3], [2:3], [2:4], [3:4] and [3:5]) are important to be considered while estimating gestational age and these ratios have a role in correctly estimating the physiological development of the fetus. So based on these findings, all the previously mentioned ratios were selected in this study to see the impact of FBSes on this maternal-fetal heartbeats coupling.

### Maternal respiration rate

In order to estimate the maternal respiration rate from the recorded ECG signal, the method introduced in [39] was adopted. The HR was determined for each R-R interval. After calculating the HR values for each R-R interval, a cubic spline interpolation technique was used to obtain the ECG Derived Respiration (EDR) waveform from HRV. Then, the Fast Fourier Transform (FFT) was applied, and the frequency with the highest amplitude within a frequency range of 0.1 to 0.5 Hz was identified as the respiration frequency [40].

### Criteria for fetal state analysis

The FBSes analysis criteria are given in Table 1 below. This is developed based on FBSes estimation, which was reported by [8, 41]. Quiet state is a state without fetal movements, which includes 1F and 3F, whereas active state is a state with fetal movements, which includes 2F and 4F. The fetal state is analyzed by studying the HRP pattern. As discussed in Table 1, if the fetal HR baseline is below 160 bpm and no acceleration in fetal HR, then it is a quiet state. Also, if the oscillation bandwidth is between < 7.5 bpm and > -7.5 bpm, it is a quiet state. If the fetal HR baseline is above 160 bpm and has accelerations > 15 bpm in fetal HR, then it is an active state. Also, if the oscillation bandwidth is between ≥ 7.5 bpm and ≤ -7.5 bpm, it is an active state.

**Table 1:** Fetal state classification criteria based on fetal HR signal [16].

Criterion	Quiet State	Active State
Baseline	< 160 bpm	> 160 bpm
Oscillation Bandwidth	<7.5 bpm and >-7.5 bpm	≥ 7.5 bpm and ≤ -7.5 bpm
Accelerations	None	> 15 bpm

### Statistical analysis

Non-parametric statistical methods to assess the mean value of λ across distinct maternal-fetal ratios within various GAs. Specifically, the Kruskal-Wallis (KW) test was utilized to examine the MFHRC across early, mid, and late GAs [17] with FBSes, with a significance threshold set at *p* < 0.05. Post hoc tests, such as Dunn’s test, with false discovery rate (FDR) correction, were employed to investigate the specific group differences further.

## Results

**Fig 2:**
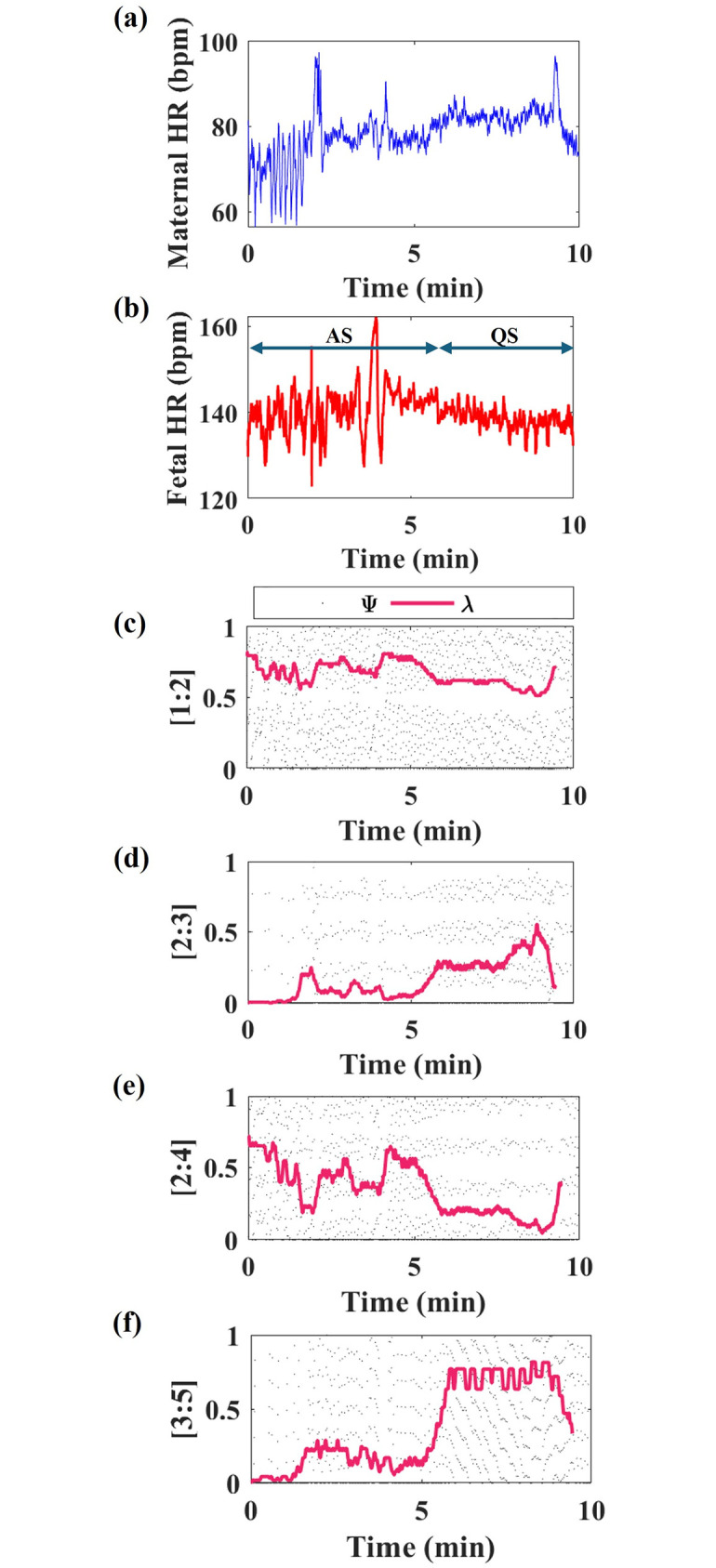
Illustration of a 37 week pregnant woman, featuring: (a) Maternal HR, (b) Fetal HR, phase coupling index (λ) at different ratios, including: (c) [1:2], (d) [2:3], (e) [2:4], and (f) [3:5]. Units: bpm (beats per minute) and min (minutes). AS denotes the Active State, while QS represents the Quiet State.

Fig 2 illustrates maternal and fetal HR time series signals for 10 min. Also, the coupling patterns between the maternal and fetal HR are characterized by λ at a ratio of [1:2], [2:3], [1:3], [2:4], and [3:5], corresponding to Fig 2(a)–2(e), respectively.

From Fig 2, it can be seen that the λ varies during the fetal active and quiet states. The fluctuations in λ at different ratios indicate changes in the degree of synchronization or coordination between the maternal and fetal HR in different fetal states.

**Fig 3:**
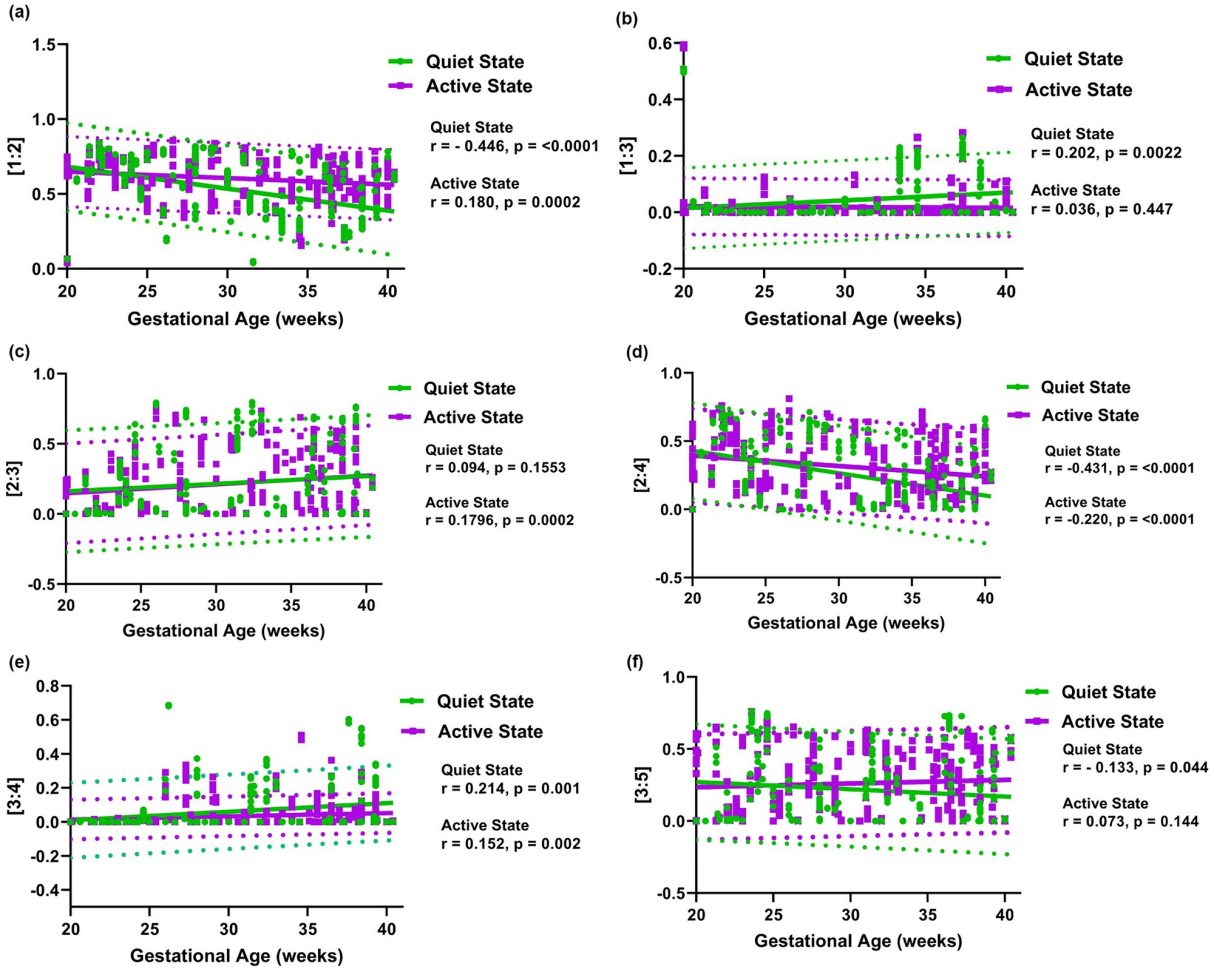
Scatterplots of the phase coupling index (λ) associated with different maternal-fetal [*m:n*] heartbeat ratios of (a) [1:2], (b) [1:3], (c) [2:3], (d) [2:4], (e) [3:4], and (f) [3:5] for GA (weeks). The 95% prediction intervals are shown as dotted lines.

Fig 3 displays scatter plots of all MFHRC ratios versus GA for different FBSes. The results indicate that the λ ratio of [1:2], [2:4], and [3:5] exhibit a similar trend of negative correlation with GA when the fetus is in a quiet state. In contrast, the λ ratio of [1:3] and [3:4] demonstrate a positive correlation with GA when the fetus is in a quiet state. The λ ratio of [2:3] also shows a positive correlation, but without any statistical significance, when the fetus is in a quiet state. On the other hand, only the λ ratio of [2:3] and [3:4] display a mild positive correlation with GA when the fetus is in an active state. Also, the λ ratio of [3:5] shows a mild positive correlation, though it is statistically insignificant. Conversely, the λ ratio of [1:2] and [2:4] show a mild negative correlation when the fetus is in an active state. The λ ratio of [1:3] remains the same throughout the GA when the fetus is in an active state.

**Fig 4:**
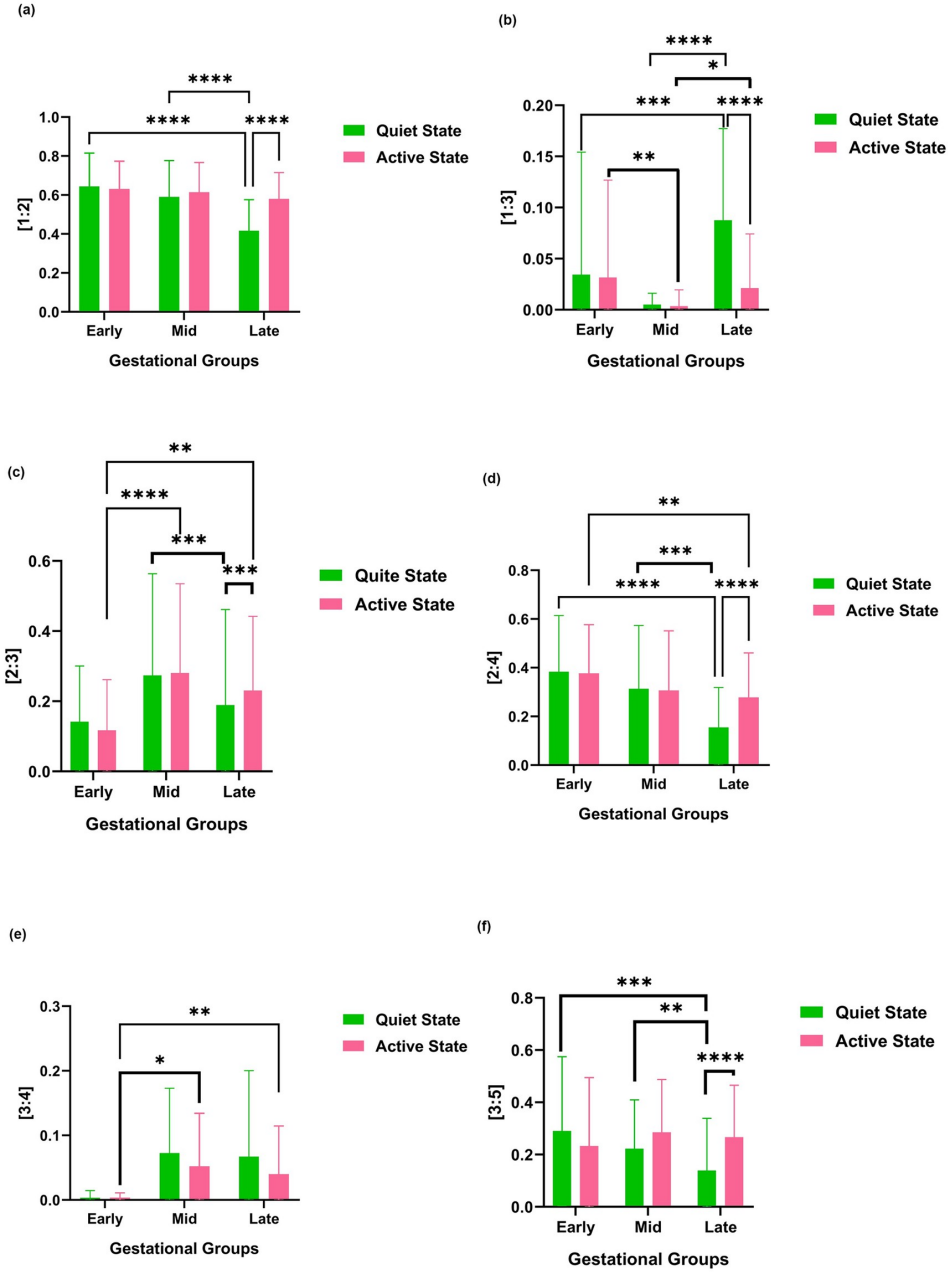
Phase coupling index with different maternal to fetal [*m:n*] ratios of (a) [1:2], (b) [1:3], (c) [2:3], (d) [2:4], (e) [3:4], and (f) [3:5] for different age groups is shown. Statistically significant variations, as determined by the Kruskal-Wallis (KW) test together with post-hoc Dunn’s test, are indicated by (*), (**), (***), and (****) for FDR-corrected p-values of <0.05, <0.01, <0.001, and <0.0001, respectively.

The findings from the box plot in Fig 4 suggest that the mean λ values for different age groups significantly vary for certain MFHRC ratios. Specifically, for the λ ratios of [1:2], [2:4], and [3:5], a significant decrease in coupling was observed from early and mid to late gestation when the fetus was in a quiet state. For the λ ratios of [2:3], a significant decrease in coupling was observed from mid to late gestation, and for the λ ratio of [1:3], λ significantly increased from early to late gestation and mid to late gestation when the fetus is in a quiet state. The λ ratio of [3:4] showed an increasing trend, though statistical significance is not shown throughout the GA in the quiet state. For the λ ratios of [1:2] and [3:5], a mild decrease in coupling was observed from mid to late gestation, though statistical significance was not shown when the fetus was in an active state. On the other hand, the mean λ significantly decreased from early to mid-gestation when the fetus is in an active state for the λ ratio of [1:3], and the mean λ significantly decreased from early to late gestation when the fetus is in an active state for the λ ratio of [2:4]. In the λ ratio of [2:3], a significant increase in coupling was observed from early to mid and late gestation when the fetus was in an active state. In contrast, a significant decrease in coupling was observed from mid to late gestation when the fetus was in a quiet state. These findings show that as GA progresses, there is a significant change in maternal-fetal heartbeat coupling, especially in late gestation, and it is interesting to see that the trend varies for each FBSes.

**Fig 5:**
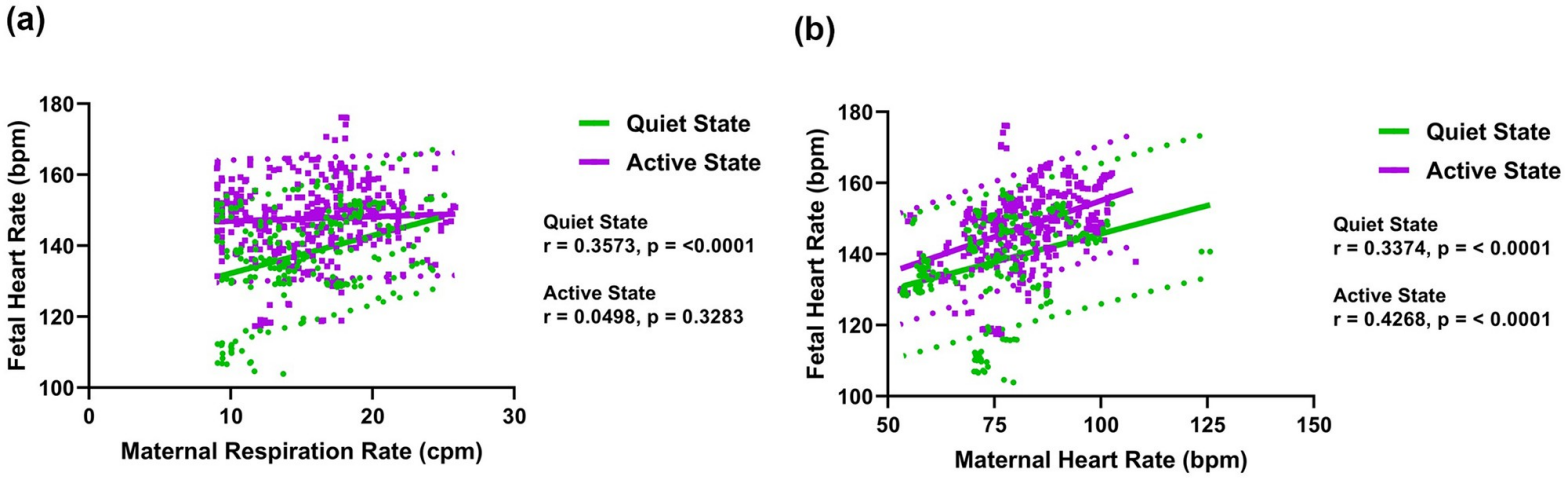
(a) Scatterplot of the fetal heart rate versus maternal respiration rate, and (b) scatterplot of the fetal heart rate versus maternal heart rate. The 95% prediction intervals are shown as dotted lines.

**Fig 6:**
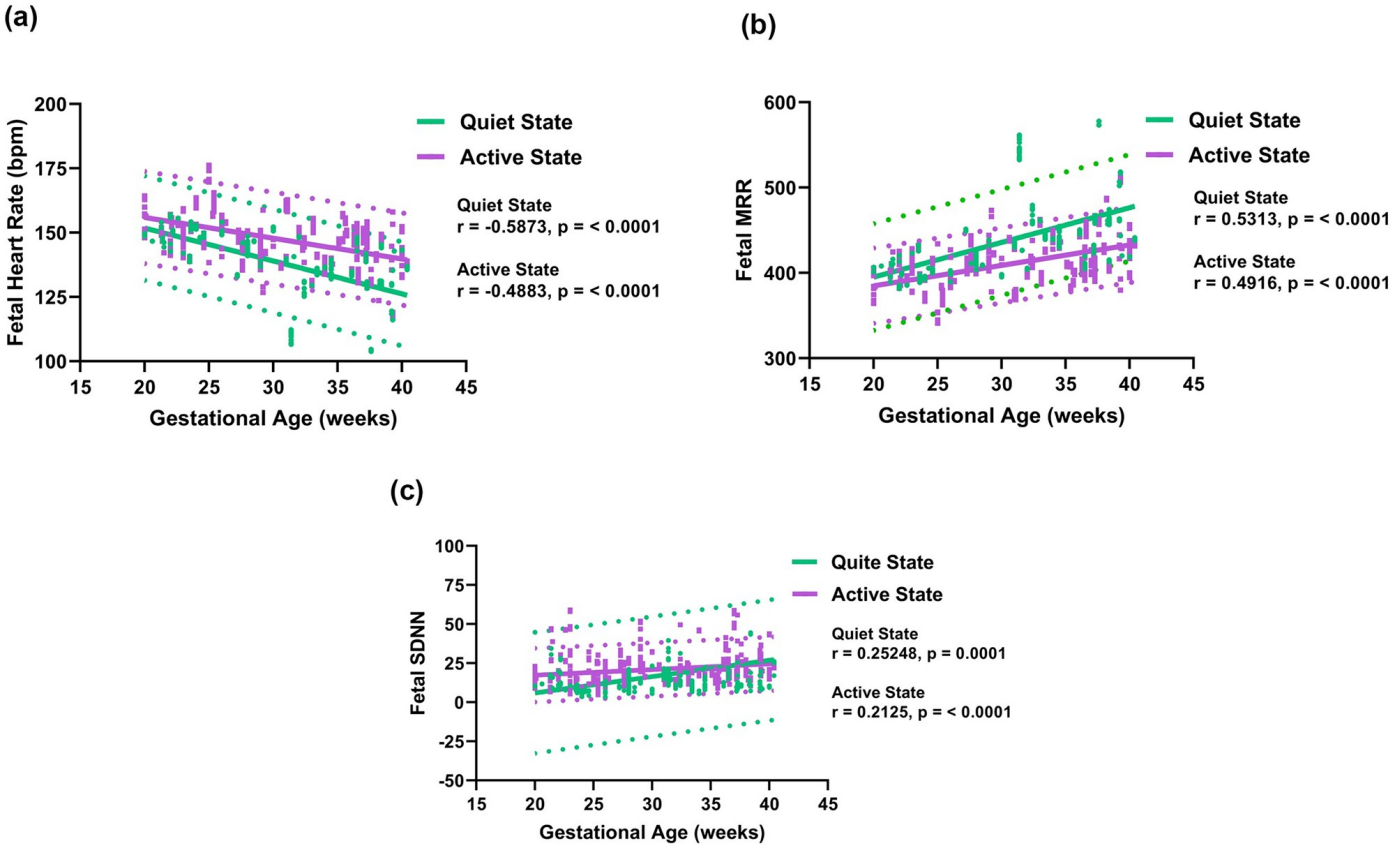
(a) Scatterplots of the fetal heart rate (bpm) versus gestational age (weeks). (b) Scatterplots of the fetal mean RR interval (MRR) versus gestational age (weeks). (c) Scatterplots of the fetal standard deviation (SDNN) versus gestational age (weeks). The 95% prediction intervals are shown as dotted lines.

Fig 5 shows the correlation between fetal HR, maternal respiration rate, and maternal HR. Fetal HR shows a positive correlation with maternal respiration rate when the fetus is in a quiet state, while no such correlation is observed when the fetus is in an active state. Fetal HR positively correlates with maternal HR, irrespective of the FBSes. Fig 6 shows the scatter plot of fetal HR, mean RR interval (MRR), and standard deviation (SDNN) versus GA, respectively. The figure shows that the active state is characterized by a lower MRR when compared to the quiet state. On the other hand, the fetal HR and SDNN values for the quiet state were significantly lower compared to the active state.

**Fig 7:**
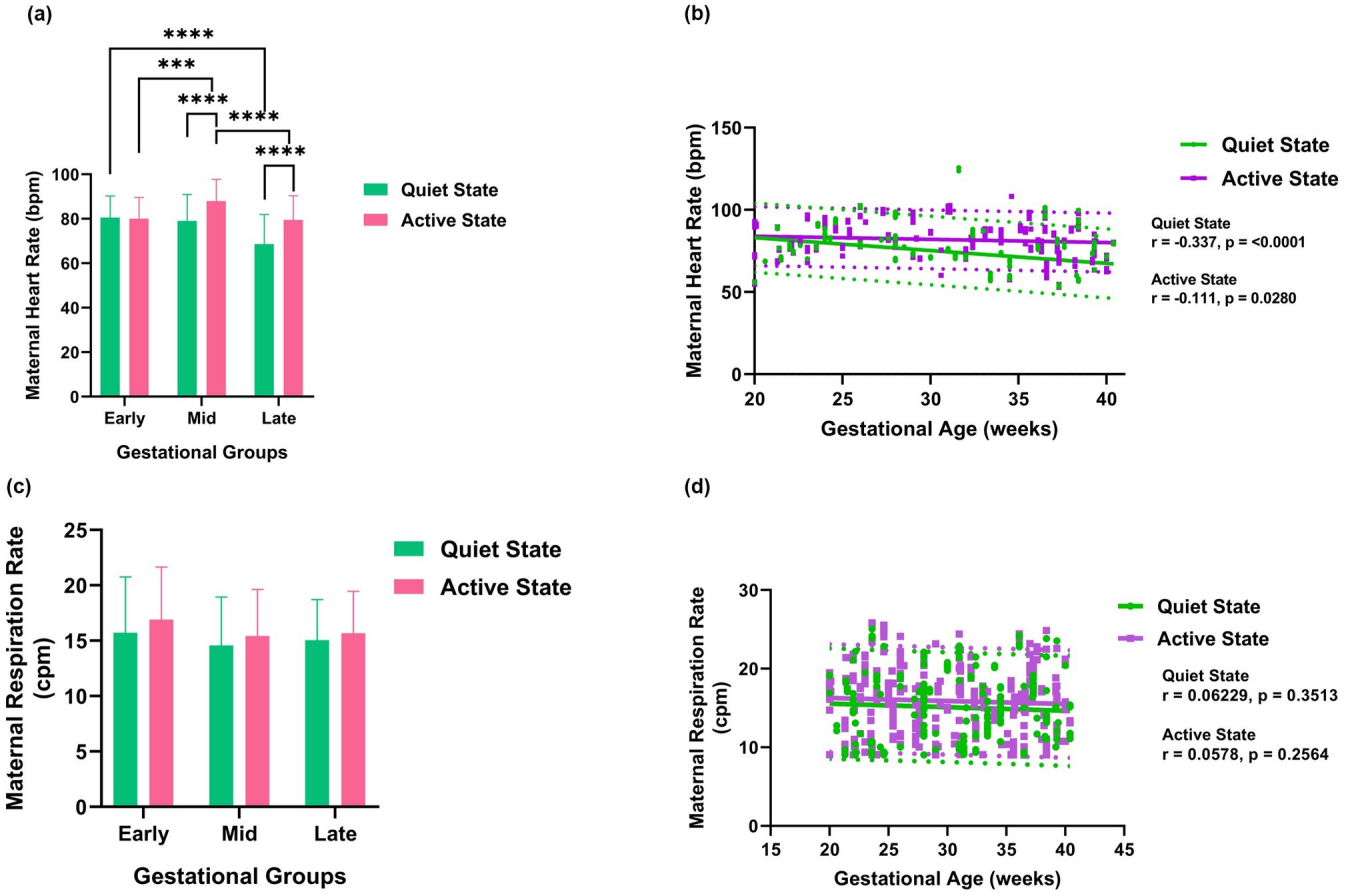
(a) Comparison of the mean maternal heart rate for different age groups. (b) Scatterplot of the maternal heart rate (bpm) versus gestational age (weeks). (c) Comparison of the mean maternal respiration rate for different age groups. (d) Scatterplot of the maternal respiration rate (cpm) versus gestational age (weeks). Significant differences according to the comparison by Kruskal-Wallis (KW) test together with post-hoc Dunn’s test, are indicated by (*), (**), (***), and (****) for FDR-corrected p-values of <0.05, <0.01, <0.001, and <0.0001, respectively. Dotted lines represent 95% prediction interval.

The box plot and scatterplot findings in Fig 7(a) and 7(b), respectively, suggest that the mean maternal HR for different age groups varies significantly. Fig 7(a) utilized the Kruskal-Wallis test with Dunn’s multiple comparisons with FDR correction to assess differences in maternal heart rate across different gestational age groups. A significant decrease in maternal HR was observed when the fetus was in quiet state from early to late gestation. A significant increase was observed from the early to the mid gestational age group, followed by significant decreases from the mid to the late gestational age group in active state. Fig 7(b) shows the scatterplot of maternal heart rate versus gestational age with a regression line for both states and a significant negative correlation with a p-value of 0.02 is observed in the active state. This mild negative correlation obtained in the scatterplot is because in the mid-gestation, there is an increase in the maternal heart rate, and it drops again when it reaches late gestation, which is observed in Fig 7(a). A significant negative correlation is also observed in the quiet state as well. The mean maternal respiration rate (Fig 7(c) and 7(d)) showed a decreasing trend from early to mid and then an increasing trend from mid to late throughout the gestational period though statistical significance is not shown, irrespective of the fetal states. The average maternal respiration rate, as depicted in Fig 7(c) and 7(d), exhibited a decline from early to mid-gestation, followed by a subsequent increase from mid to late gestation. Although the statistical significance wasn’t shown, this pattern is observed throughout the entire pregnancy.

**Fig 8:**
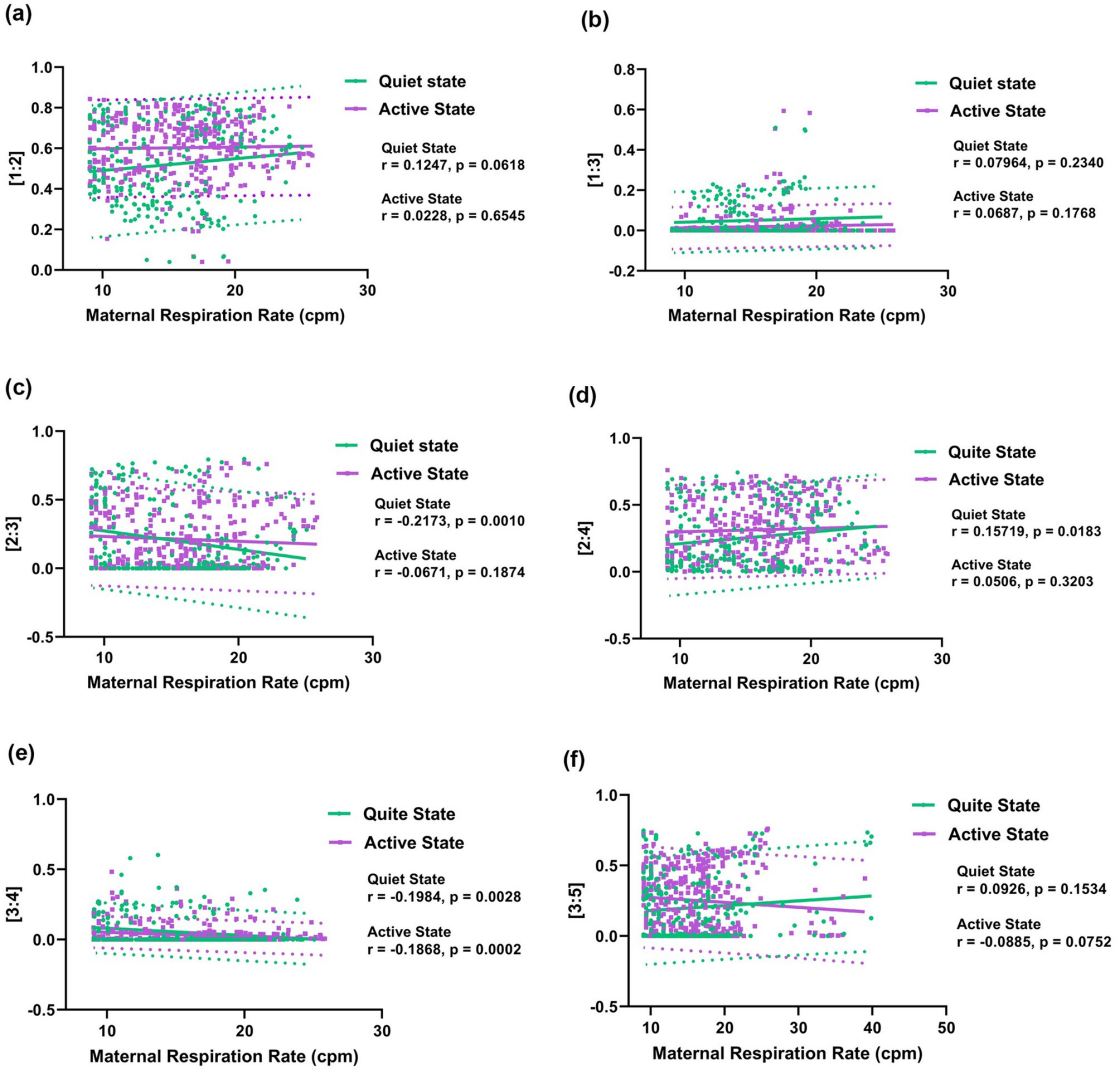
Scatterplots of the phase coupling index (λ) associated with different maternal-fetal [*m:n*] heartbeat ratios of (a) [1:2], (b) [1:3], (c) [2:3], (d) [2:4], (e) [3:4], and (f) [3:5] with respect to maternal respiration rate. The 95% prediction intervals are shown as dotted lines.

Fig 8 displays scatter plots of all MFHRC ratios versus maternal respiration rates for different FBSes. The results indicate that the λ ratio of [2:4] exhibits a trend of positive correlation with maternal respiration rate when the fetus is in a quiet state, while the λ ratio of [2:3] and [3:4] demonstrate a negative correlation with maternal respiration rate when the fetus is in a quiet state. Also, the λ ratio of [1:2] shows a mild positive correlation, though the statistical significance is not there. On the other hand, in the active state, the λ ratios of [1:2], [1:3], and [2:4] show a mild positive correlation, though the statistical significance is not there. The λ ratio of [3:4] shows a significant negative correlation when the fetus is in an active state. All the other ratios showed mild negative correlation, though statistical significance is not there.

## Discussion

### 1. Development of maternal-fetal cardiac coupling

Evaluating fetal heart growth throughout pregnancy holds significant importance in ensuring the healthy development of the fetus’s physical and psychological aspects. Furthermore, conducting this assessment in conjunction with monitoring the maternal cardiac changes during gestation is essential, as research indicates that these changes can profoundly impact the development of the fetal cardiac system. Previous studies have shown that incorporating maternal-fetal cardiac coupling improved the GA estimation [25, 26].

In this study, we examined whether there is a connection between the synchronization of maternal and fetal heart rhythms during pregnancy and fetal behavioral patterns. We conducted this study with the understanding that the fetal HRP may influence changes in the MFHRC. Our study shows that FBSes influence MFHRC, particularly during late gestation (Fig 4). In late gestation, there is a significant change observed in MFHRC according to FBSes in all ratios. The significant influence of FBSes on MFHRC was observed mainly during late gestation and not in the earlier stages of pregnancy.

Different coupling scenarios, such as the λ ratio of [1:2], [2:4] and [3:5] decrease from early to late gestation. This indicates that proper fetal cardiac development leads to higher fetal HR than maternal HR and stabilization of fetal heart function in late gestation [36, 42]. In our study, λ ratios of [1:2] and [2:4] followed the same trend in both FBSes, while for the λ ratio of [3:5], only a quiet state followed this trend. It is hypothesized that constant patterns of HRP associated with FBSes lead to proper cardiac development. On the other hand, the λ ratio of [1:3] and [3:4] showed an increasing pattern with GA, indicating more fetal heart activity closer to late pregnancy [26]. This change in the coupling in late gestation for different FBSes shows that as the fetus matures, it becomes more independent of the mother for its fetal HRV and ANS development. According to a previous study, age-dependent growth is visible in the quiet state by increasing the sympathetic and vagal control [43]. In our study, age-dependent change in MFHRC is present in quiet states and active states, which indicates that FBSes have an impact on MFHRC especially in late gestation. It is hypothesized that differences in maternal oxygen and nutrient transfer, changes in the placental flow, changes in maternal blood pressure, and also hormonal levels with different FBSes might be the underlying reason behind the impact on MFHRC in late gestation.

### 2. Roles of maternal respiration on fetal behavior

We have also investigated whether maternal respiration plays a role in influencing fetal behavior and MFHRC. Previous studies reported that higher MFHRC happens at a higher maternal respiratory rate [22, 24]. In our study, we observed higher synchronization at a higher maternal respiration rate in quiet state in some coupling ratios. We found significant negative correlations between maternal respiration rate and maternal-fetal synchronization in the [3:4] ratio in the fetal quiet state. We also found that this significant correlation, either positive or negative, happens between MFHRC and maternal respiration rate in the fetal quiet state in all λ ratios, though the statistical significance is not shown in ratios [1:2], [1:3], and [3:5]. All MFHRC ratios, except for [3:4], exhibited no significant correlation with maternal heart rate in the fetal active state.

### 3. Potential clinical implications

The study suggests that the MFHRC positively correlates with the maternal breathing rate, and effective communication between mother and fetus happens in a fetal quiet state. In studies with adults, it has been shown that when people are exposed to a gentle, rhythmic sound that matches the rhythm of their heartbeat, their HR can sync up with that sound [44]. By the middle of pregnancy, the fetus’s ears and hearing are developed enough to detect sounds. It is possible that the mother’s heart, which is beating in its rhythm, could affect the fetus’s heart rhythm. This effect might depend on how fast the mother is breathing. When the mother breathes quickly, her heartbeats are closer together, and this might create conditions where the fetus’s heart can more easily match its rhythm to the mother’s. Essentially, it is like the fetus’s heart is following the beat of the mother’s heart, and faster breathing by the mother might make this synchronization more likely to happen. The findings of this study suggest MFHRC changes are more noticeable during quiet states, especially in late gestation. This highlights the maturation of the fetus.

The fetus’s heart can change its HR and HRV in response to external events, and FBSes play an important role in tuning it. However, we still need to do more research to understand exactly how the mother’s heart affects the fetus’s heart, incorporating all four FBSes in late gestation. By figuring out how these two separate heart systems work together in different situations, we can learn more about how a fetus develops before birth and how mothers and fetuses communicate even before birth. This knowledge could help us better monitor pregnancies and detect potential problems early on.

While our research revealed the relationship between MFHRC and FBSes, it’s important to acknowledge certain limitations. Though we successfully demonstrated the relationship, it’s worth noting that we couldn’t take all four FBSes due to the lack of a larger dataset. Also, our dataset lacked abnormal mothers and fetuses. In order to deepen our comprehension of the patient-specific coupling mechanisms throughout GA, it is imperative to conduct a subsequent study that follows the same individuals from early to late GA. This method would facilitate a more thorough examination on an individual basis and offer greater insights into the evolution of these mechanisms over the course of time.

## Conclusions

The study investigated the impact of FBSes on MFHRC at different stages of gestation. The results showed that during late gestation, FBSes influenced the coupling between maternal and fetal heartbeats. When the fetus was in a quiet state, the study observed a noteworthy increase in the MFHRC ratio of [2:4] during higher respiratory rates and in late gestation. This suggests a correlation between the MFHRC and the respiratory rate of the mother. These findings emphasize the influence of FBSes, particularly in late gestation, on MFHRC. Future works include the incorporation of large datasets for validating the study and also considering all four FBSes. Additionally, maternal-fetal coupling in abnormal fetuses, taking FBSes into consideration, could be investigated. Furthermore, conducting an in-depth investigation focusing on individual patients over the course of gestation would contribute to a deeper understanding of the MFHRC and fetal maturation. Evaluating the extent of this coupling at various GAs could be crucial in identifying new clinical indicators of normal prenatal development and deviations from it.
